# Supplementary material for: Structural basis for importin alpha 3 specificity of W proteins in Hendra and Nipah viruses
Source: Nat Commun. 2018 Sep 12;9:3703. doi: 10.1038/s41467-018-05928-5 (PMC6135763; doi:10.1038/s41467-018-05928-5)
Supplement: Supplementary file 1 — Supplementary Information [file 41467_2018_5928_MOESM1_ESM.pdf]

Supplementary information for Structural basis for importin alpha 3 binding specificity of W proteins in Hendra and Nipah viruses

Smith et al.

Supplementary Figure 1 (A) The Henipavirus W proteins are encoded by the Phosphoprotein (P)-gene through mRNA editing. Of the four protein products, W contains a unique C-terminus NLS that is responsible for mediating translocation to the nucleus. (B) Human importin α isoforms are grouped into three αSFs (left)[1,2], with the sequence identity of each importin α isoform ranging from 42-51% between subfamilies.

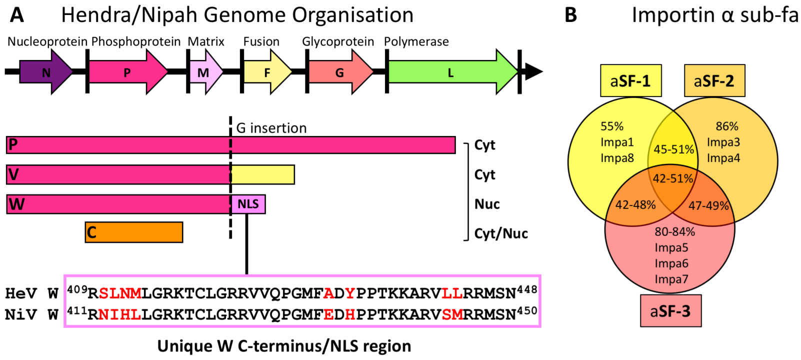

%	α1	α8	α3	α4	α5	α6	α7
α1	100	55	51	50	46	48	48
α8	55	100	45	45	42	42	42
α3	51	45	100	86	47	48	48
α4	50	45	86	100	48	49	48
α5	46	42	47	48	100	80	81
α6	48	42	48	49	80	100	84
α7	48	42	48	48	81	84	100

Supplementary Figure 2

Full, uncropped Western blots presented in the manuscript. Boxes with dash lines indicate regions displayed in the main article.

(A) Related to Figure 1B

Figure S2a (related to Figure 1B)

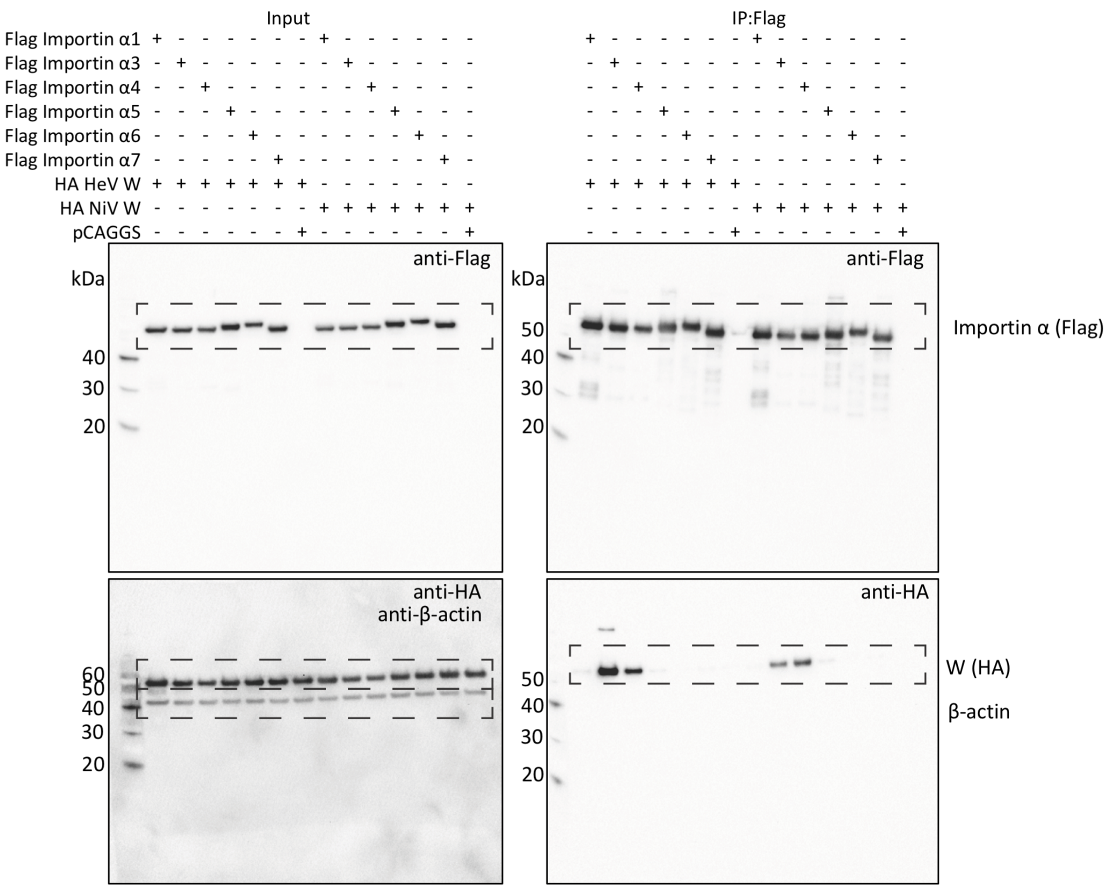

(B) Related to Figure 4B

Figure S2b (related to Figure 4B)

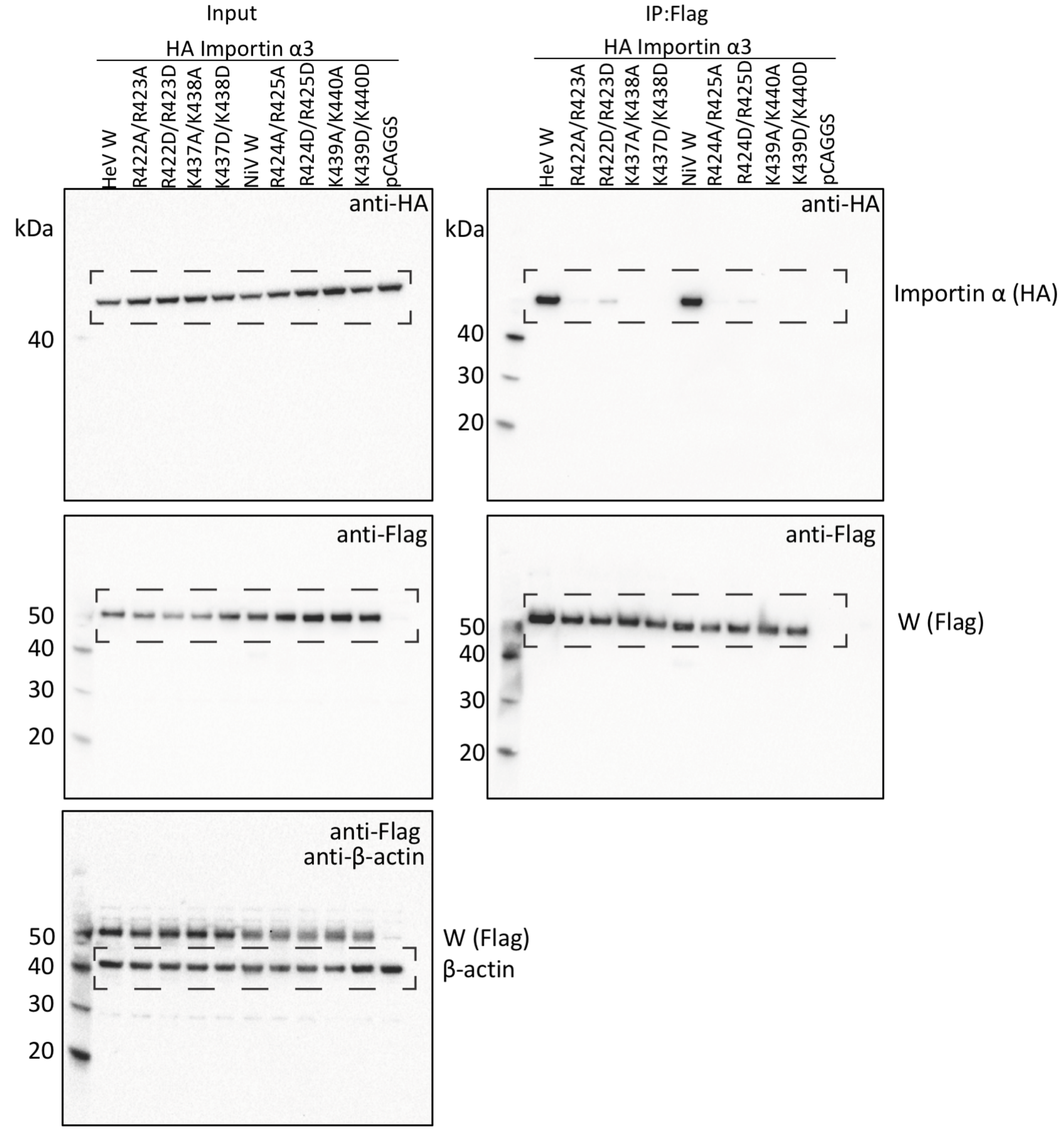

(C) Related to Figure 4C

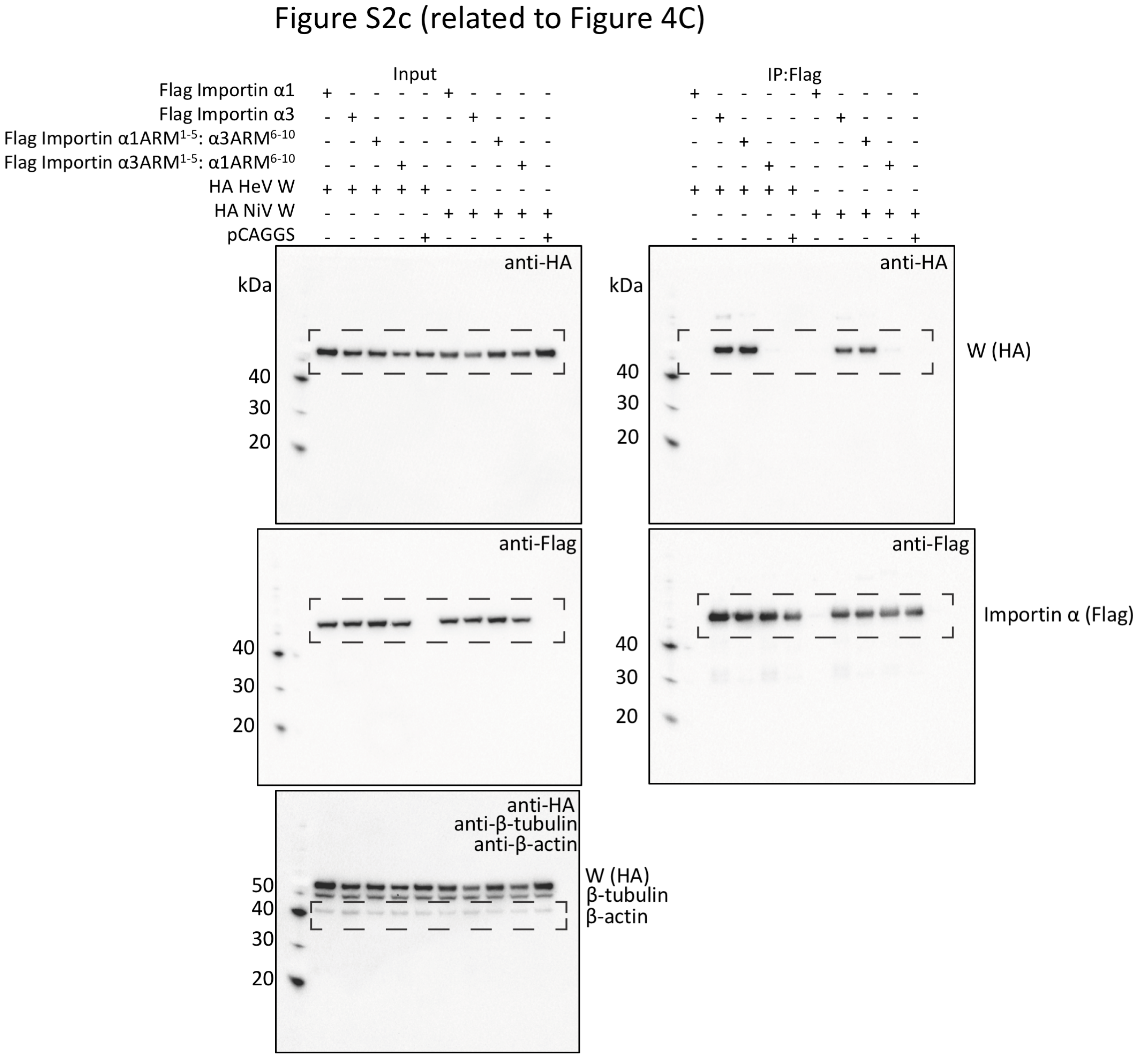

(D) Related to Figure 7C

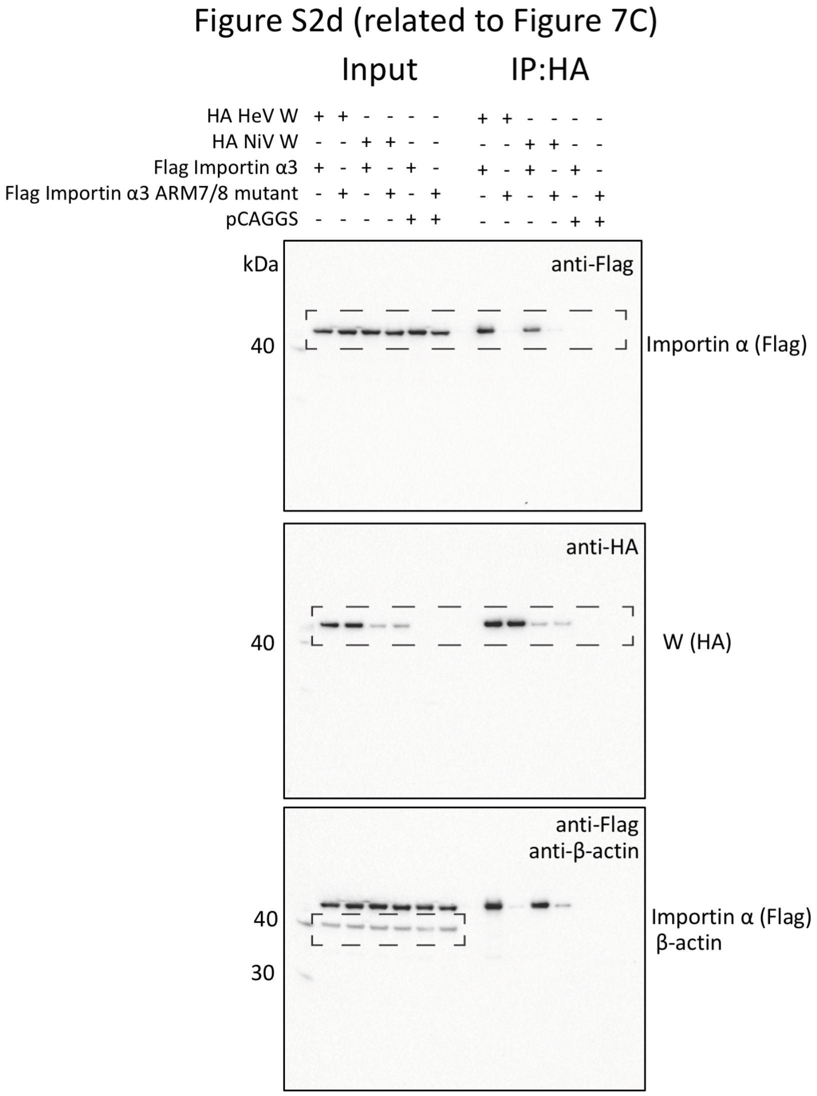

Supplementary Figure 3

Structural superposition of the three Importin α3 structures solved in this study.

Importin α3:HeV W crystal form 1 (see Table 1) was used as the reference structure (coloured orange), and crystal forms 2 and 3 were superimposed in UCSF Chimera using MatchEnsemble. α-helices are represented as cylinders, and coloured blue for conservation, and red for r.m.s.d variances above 2.5 Å. The r.m.s.d. plot was generated using MatchAlign, and MatchAssess functions.

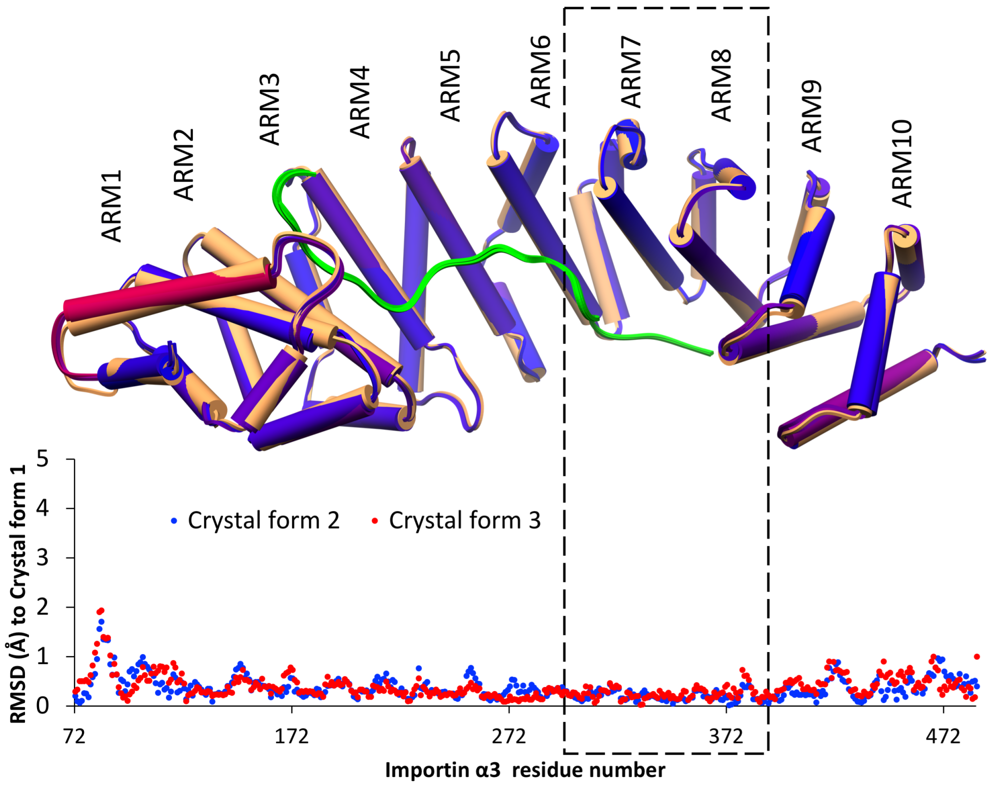

Supplementary Figure 4

Sequence alignment of importin α1 and α3. ClustalW alignment of importin α1, P52293, and importin α3, O00629, with hydrogen bonded residues of importin α3 with W protein denoted with blue asterisk and salt bridge interaction residues between importin α3 and W protein denoted with a red asterisk.

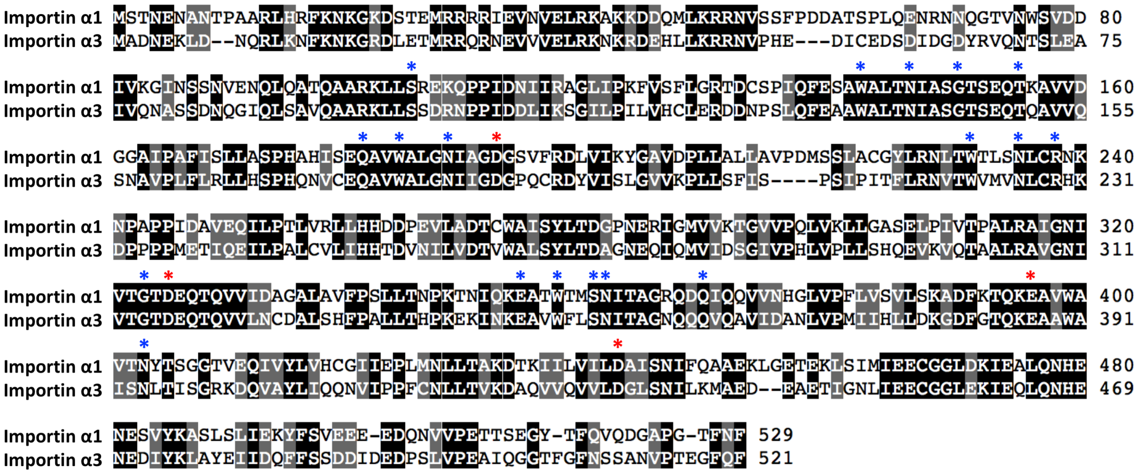

Supplementary Figure 5

ELISA binding data for the Importin α1$^{ARM1\text{-}5}$:α3$^{ARM6\text{-}10}$ gain of function chimera performed with anti-6x HIS 4HRP conjugated antibody and developed using TMB substrate, with HeV W, NiV W and GST as a control.

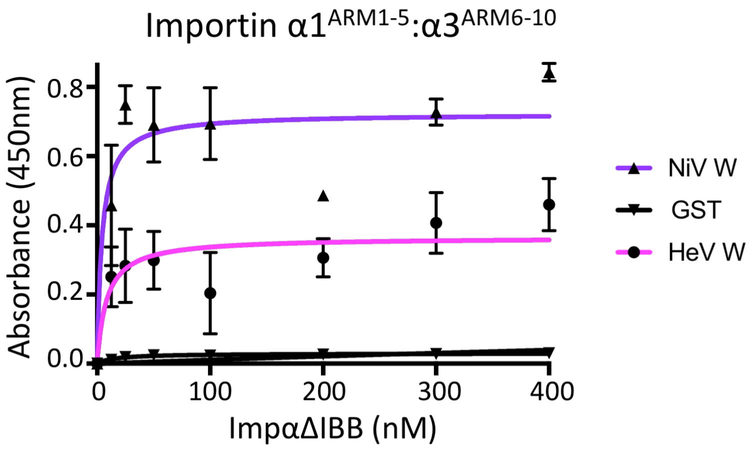

Supplementary Figure 6. (A) Uncropped gel of the GST SV40 T-ag pulldown assay confirming Importin $\alpha 1^{ARM1\text{-}5}$:$\alpha 3^{ARM6\text{-}10}$ chimera and Importin α3 ARMs 7/8 (348A/N352A/E387A/N394A) mutant are functionally active and bind the GST:SV40-Tag NLS. GST alone is included as a negative control. (B) Uncropped gel showing the input proteins used in (A).

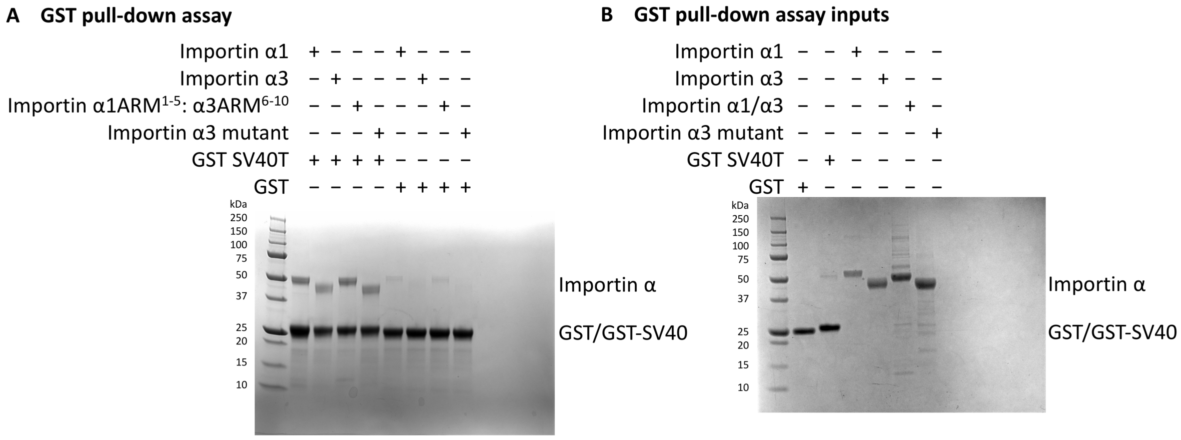

Supplementary Table 1: ELISA binding data for W protein mutants and importins

K_D (nM)				
GST-NLS	Importin α1	Importin α3	Importin α7	Importin $\alpha1^{ARM1-5}:\alpha3^{ARM6-10}$
GST-NiV W	1,495 ± 489	14.4 ± 2.5	1,147 ± 196.7	4.3 ± 3.4
GST-HeV W	1,363 ± 211	19.9 ± 2.7	1,468 ± 238.3	8.3 ± 7.5
GST-R422A/R423A	-	2,208 ± 354	-	-
GST- K437A/K438A	-	>10,000	-	-
GST-R422A/R423A/K437A/K438A	-	>10,000	-	-

Supplementary Table 2: MST binding data for HeV W and importins

	K_D (nM) Repeat 1	K_D (nM) Repeat 2	K_D (nM) Repeat 3
Importin α1	860	553	632
Importin α3	4.31	3.33	5.54
Importin α7	631	901	557

Supplementary Table 3: PISA interaction analysis of W Importin α1 complexes. Note: HB = hydrogen bond, SB = salt bridge

	**Importin α1**	**Distance (Å)**	**HeV W**	**Importin α1**	**Distance (Å)**	**NiV W**
HB 1	ARG 238[NH2]	3.39	PRO 434[O]	ARG 238[NH2]	3.19	PRO 436[O]
HB 2	ARG 238[NH1]	3.45	PRO 434[O]	ARG 238[NH1]	3.07	PRO 436[O]
HB 3	TRP 231[NE1]	3.32	PRO 435[O]	TRP 231[NE1]	3.17	PRO 437[O]
HB 4	ASN 235[ND2]	2.91	THR 436[O]	ASN 235[ND2]	2.95	THR 438[O]
HB 5	TRP 184[NE1]	2.84	LYS 438[O]	TRP 184[NE1]	2.88	LYS 440[O]
HB 6	ASN 188[ND2]	3.08	LYS 438[O]	ASN 188[ND2]	3.03	LYS 440[O]
HB 7	TRP 142[NE1]	3.00	ARG 440[O]	TRP 142[NE1]	2.95	ARG 442[O]
HB 8	ASN 146[ND2]	2.97	ARG 440[O]	ASN 146[ND2]	2.84	ARG 442[O]
HB 9	GLY 150[O]	2.91	LYS 437[NZ]	GLY 150[O]	3.10	LYS 439[NZ]
HB 10	THR 155[OG1]	2.82	LYS 437[NZ]	THR 155[OG1]	2.88	LYS 439[NZ]
HB 11	ASP 192[OD1]	3.00	LYS 437[NZ]	ASP 192[OD1]	3.04	LYS 439[NZ]
HB 12	ASN 188[OD1]	2.84	LYS 438[N]	ASN 188[OD1]	2.85	LYS 440[N]
HB 13	ASN 228[OD1]	3.05	LYS 438[NZ]	ASN 228[OD1]	3.12	LYS 440[NZ]
HB 14	ASN 146[OD1]	2.84	ARG 440[N]	ASN 146[OD1]	2.83	ARG 442[N]
HB 15	GLN 181[OE1]	2.70	ARG 440[NH1]	GLN 181[OE1]	2.75	ARG 442[NH1]
SB 1	ASP 192[OD1]	3.00	LYS 437[NZ]	ASP 192[OD1]	3.04	LYS 439[NZ]

Supplementary Table 4: PISA interaction analysis of W Importin α3 complexes. Note: HB = hydrogen bond, SB = salt bridge

	Importin α3	Distance (Å)	HeV W	Importin α3	Distance (Å)	NiV W
HB 1	ASP 433[OD2]	3.64	CYS 419[N]	ASN 394[ND2]	3.14	GLY 423[O]
HB 2	ASN 394[OD1]	2.93	GLY 421[N]	ASN 352[ND2]	3.08	ARG 425[O]
HB 3	ASN 352[O]	2.80	ARG 422[NE]	TRP 348[NE1]	3.30	ARG 425[O]
HB 4	ASP 316[OD1]	2.81	ARG 422[NH1]	TRP 348[NE1]	3.36	VAL 426[O]
HB 5	GLY 314[O]	2.97	ARG 422[NH1]	ARG 229[NH1]	2.89	PRO 436[O]
HB 6	GLN 360[OE1]	3.18	ARG 422[NH2]	ARG 229[NH2]	3.47	PRO 437[O]
HB 7	ASN 352[O]	3.14	ARG 422[NH2]	TRP 222[NE1]	2.74	PRO 437[O]
HB 8	ASN 352[OD1]	2.89	ARG 423[N]	ASN 226[ND2]	3.01	THR 438[O]
HB 9	SER 351[OG]	2.96	ARG 423[NH2]	TRP 179[NE1]	2.94	LYS 440[O]
HB 10	GLU 387[OE2]	2.53	ARG 423[NH2]	ASN 183[ND2]	2.92	LYS 440[O]
HB 11	GLU 345[OE1]	2.88	PHE 430[N]	TRP 137[NE1]	2.80	ARG 442[O]
HB 12	THR 150[OG1]	2.84	LYS 437[NZ]	ASN 141[ND2]	2.85	ARG 442[O]
HB 13	ASP 187[OD1]	2.72	LYS 437[NZ]	SER 100[OG]	3.79	SER 444[OG]
HB 14	GLY 145[O]	3.03	LYS 437[NZ]	ASN 394[OD1]	2.95	GLY 423[N]
HB 15	ASN 183[OD1]	2.83	LYS 438[N]	ASN 352[O]	2.85	ARG 424[NE]
HB 16	ASN 141[OD1]	2.94	ARG 440[N]	ASP 316[OD1]	2.77	ARG 424[NH1]
HB17	GLN 176[OE1]	2.87	ARG 440[NH1]	GLY 314[O]	2.86	ARG 424[NH1]
HB 18	SER 100[O]	2.80	LEU 442[N]	GLN 360[OE1]	3.14	ARG 424[NH2]
HB 19	ASN 394[ND2]	3.28	GLY 421[O]	ASN 352[OD1]	2.94	ARG 425[N]
HB 20	ASN 352[ND2]	3.06	ARG 423[O]	GLU 387[OE1]	3.15	ARG 425[NH1]
HB 21	TRP 348[NE1]	3.14	ARG 423[O]	SER 351[OG]	3.13	ARG 425[NH2]
HB 22	TRP 348[NE1]	3.22	VAL 424[O]	GLU 387[OE2]	2.46	ARG 425[NH2]
HB 23	ARG 229[NH1]	2.84	PRO 434[O]	GLU 345[OE1]	2.96	PHE 432[N]
HB 24	ARG 229[NH2]	3.11	PRO 434[O]	THR 150[OG1]	2.96	LYS 439[NZ]
HB 25	ARG 229[NH2]	3.38	PRO 435[O]	ASP 187[OD1]	2.83	LYS 439[NZ]
HB 26	TRP 222[NE1]	2.84	PRO 435[O]	GLY 145[O]	3.14	LYS 439[NZ]
HB 27	ASN 226[ND2]	2.95	THR 436[O]	ASN 183[OD1]	2.81	LYS 440[N]
HB 28	TRP 179[NE1]	2.90	LYS 438[O]	ASN 141[OD1]	2.91	ARG 442[N]
HB 29	ASN 183[ND2]	2.93	LYS 438[O]	GLN 176[OE1]	2.76	ARG 442[NH1]
HB 30	TRP 137[NE1]	2.90	ARG 440[O]	SER 100[O]	2.92	SER 444[N]
HB 31	ASN 141[ND2]	2.90	ARG 440[O]	SER 100[O]	3.51	SER 444[OG]
SB 1	ASP 433[OD2]	3.64	CYS 419[N]	ASP 316[OD1]	2.77	ARG 424[NH1]
SB 2	ASP 316[OD1]	2.81	ARG 422[NH1]	ASP 316[OD1]	3.26	ARG 424[NH2]
SB 3	ASP 316[OD1]	3.36	ARG 422[NH2]	GLU 387[OE1]	3.15	ARG 425[NH1]
SB 4	GLU 387[OE1]	3.55	ARG 423[NH1]	GLU 387[OE2]	3.33	ARG 425[NH1]
SB 5	GLU 387[OE2]	3.09	ARG 423[NH1]	GLU 387[OE1]	3.78	ARG 425[NH2]
SB 6	GLU 387[OE2]	2.53	ARG 423[NH2]	GLU 387[OE2]	2.46	ARG 425[NH2]
SB 7	ASP 187[OD1]	2.72	LYS 437[NZ]	ASP 187[OD1]	2.83	LYS 439[NZ]

Supplementary Table 5: Overview comparison of the interaction interfaces between importin α1 and α3 for HeV and NiV W proteins

	**HeV W: Importin α1**	**HeV W: Importin α3**	**NiV W: Importin α1**	**NiV W: Importin α3**
Total buried surface area of binding interface ($Å^2$)	683.5	1,616.9	688.7	1,591.8
ARM domain interactions on importin alpha α	2-4	2-4, 6-9	2-4	2-4, 6-9
W protein residues mediating interactions	434-441	419-444	436-443	421-446
Hydrogen bond interactions	15	31	15	31
Salt bridge interactions	1	7	1	7

Supplementary Table 6: Primers used in this study

Primer name	Primer Sequence	Primer Use
NiV W fwd Not1	gcgcgcggccgcagataaattggaactag	NiV W forward primer with a NotI site
NiV/HeV W Rev Xho1	gcgcctcgagtcagttggacattctc	NiV W/HeV W reverse primer with a XhoI site
HeV W fwd Not1	gcgcgcggccgcagacaagttggatctag	HeV W forward primer with a NotI site
NiV/HeV W R422A/R423A fwd	gtgcctgggtgcagcagtggtgcaac	Generate HeV W R422A/R423A or NiV W R424A/R425A by overlapping PCR
NiV/HeV W R422A/R423A Rev	gttgcaccactgctgcacccaggcac	Generate HeV W R422A/R423A or NiV W R424A/R425A by overlapping PCR
NiV/HeV W R422D/R423D fwd	gtgcctgggtgacgacgtggtgcaac	Generate HeV W R422D/R423D or NiV W R424D/R425D by overlapping PCR
NiV/HeV W R422D/R423D rev	gttgcaccacgtcgtcacccaggcac	Generate HeV W R422D/R423D or NiV W R424D/R425D by overlapping PCR
HeV W K437A/K438A Rev Xho1	gcgcctcgagtcagttggacattctccgcagtaacactcttgctgccgcggttgg	Generate HeV W K437A/K438A with a XhoI site
HeV W K437D/K438D Rev Xho1	gcgcctcgagtcagttggacattctccgcagtaacactcttgcatcgtcggttgg	Generate HeV W K437D/K438D with a XhoI site
NiV W K439A/K440A Rev Xho1	gcgcctcgagtcagttggacattctccgcattgacactcttgccgccgcggtagg	Generate NiV W K439A/K440A with a XhoI site
NiV W K439D/K440D Rev Xho1	gcgcctcgagtcagttggacattctccgcattgacactcttgcatcgtcggtagg	Generate NiV W K439D/K440D with a XhoI site
Impα1/3 279/271 fwd	ctatttcctaccttactgatgctggcaatgaac	Generate Importin α1/Importin α3 chimera
Impα1/3 279/271 rev	gttcattgccagcatcagtaaggtaggaaatag	Generate Importin α1/Importin α3 chimera
Impα3/1 270/280 fwd	ctctcttaccttactgatggtccaaatgaac	Generate Importin α3/Importin α1 chimera
Impα3/1 270/280 rev	gttcatttggaccatcagtaaggtaagagag	Generate Importin α3/Importin α1 chimera
Impα1 Not1 fwd	gcgcgcggccgcatccaccaacgag	Importin α1 forward primer with a NotI site
Impα1 Xho1 Rev	gcgcctcgagctaaaagttaaag	Importin α1 reverse primer with a XhoI site
Impα3 Not1 fwd	gcgcgcggccgcagcggacaacgag	Importin α3 forward primer with a NotI site
Impα3 Xho1 Rev	gcgcctcgagctaaaactggaac	Importin α3 reverse primer with a NotI site
Impα3 N348A/N352A fwd	gaagcagtggcgttcctctccgccatcactgcag	Generate Impα3 ARM7/8 mutant
Impα3 N348A/N352A rev	ctgcagtgatggcggagaggaacgccactgcttc	Generate Impα3 ARM7/8 mutant
KPNA4 E387A fwd	cactcaaaaagcagctgcttggg	Generate Impα3 ARM7/8 mutant
KPNA4 E387A rev	cccaagcagctgctttttgagtg	Generate Impα3 ARM7/8 mutant
KPNA4 N394A fwd	ggccataagtgccttaacaattag	Generate Impα3 ARM7/8 mutant
KPNA4 N394A rev	ctaattgttaaggcacttatggcc	Generate Impα3 ARM7/8 mutant

Supplementary References

1 Tsuji, L., Takumi, T., Imamoto, N. & Yoneda, Y. Identification of novel homologues of mouse importin α, the α subunit of the nuclear pore-targeting complex, and their tissue-specific expression. *FEBS letters* **416**, 30-34 (1997).

2 Pumroy, R. A. & Cingolani, G. Diversification of importin-α isoforms in cellular trafficking and disease states. *Biochemical Journal* **466**, 13-28 (2015).
